# A Hierarchical Matrix Factorization-Based Method for Intelligent Industrial Fault Diagnosis

**DOI:** 10.3390/s24165408

**Published:** 2024-08-21

**Authors:** Yanxia Li, Han Zhou, Jiajia Liu, Xuemin Tan

**Affiliations:** 1 School of Automation, Chengdu University of Information Technology, Chengdu 610225, China; liyanxia106@gmail.com (Y.L.); liujj@cuit.edu.cn (J.L.); tan_xue_min@126.com (X.T.); 2 School of Automation, Chongqing University, Chongqing 400044, China

**Keywords:** industrial processes, fault diagnosis, non-negative matrix factorization, hierarchical, nonlinear

## Abstract

Data-driven fault diagnosis, identifying abnormality causes using collected industrial data, is one of the challenging tasks for intelligent industry safety management. It is worth noting that practical industrial data are usually related to a mixture of several physical attributes, such as the operating environment, product quality and working conditions. However, the traditional models may not be sufficient to leverage the coherent information for diagnostic performance enhancement, due to their shallow architecture. This paper presents a hierarchical matrix factorization (HMF) that relies on a succession of matrix factoring to find an efficient representation of industrial data for fault diagnosis. Specifically, HMF consecutively decomposes data into several hierarchies. The intermediate hierarchies play the role of analysis operators which automatically learn implicit characteristics of industrial data; the final hierarchy outputs high-level and discriminative features. Furthermore, HMF is also extended in a nonlinear manner by introducing activation functions, referred as NHMF, to deal with nonlinearities in practical industrial processes. The applications of HMF and NHMF to fault diagnosis are evaluated by the multiple-phase flow process. The experimental results show that our models achieve competitive performance against the considered shallow and deep models, consuming less computing time than deep models.

## 1. Introduction

To ensure industrial processes’ reliability and safety, fault diagnosis, which identifies abnormality causes at an early stage, is one of the ongoing research activities in intelligent industry safety management. Over the past decades, the increasing system complexity has posed challenges to traditional methods [1], since these methods usually require a good deal of expertise or rigorous system structure clarification. Meanwhile, with the extensive usage of intelligent sensors and computer systems, amounts of process data can be recorded and stored in industrial databases. Under these circumstances, data-driven methods, which automatically extract valuable information from industrial data and make reliable decisions without much prior knowledge, supply a feasible solution to the fault diagnosis problem.

For data-driven fault diagnosis, it is reasonable to treat process data that share similar features as the same fault class. If a new receiving datum is assigned to a known class that has similar features to it, its fault is then identified. One class of methodologies is collectively referred to as Multivariate Statistical Analysis (MSA), using statistical methodologies to analyze jointly two or more statistical variables collected from the industry. Basic techniques include Principal Component Analysis (PCA) [2], Fisher Discriminant Analysis (FDA) [3], Independent Component Analysis (ICA) [4], etc. Another class can be called representation learning (RL)-based methods. They learn representative features of fault data so that different faults can be distinguished by trained classifiers. Popular techniques include Dictionary Learning [5], Manifold Learning [6], Non-negative Matrix Factorization (NMF) [7,8,9,10], etc. Their advantages lie in the model interpretability and complexity reduction in analysis.

**Figure 1:**
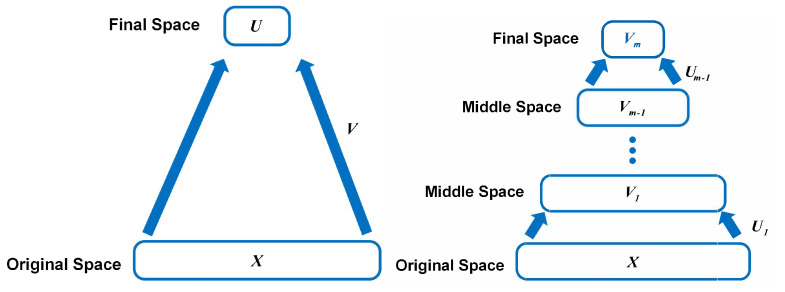
Illustration Authors: We have noted that changes to the position of figures and tables may occur during the production stage. of hypothesis of the characteristics of the shallow model and hierarchical model.

However, practical industrial process data usually contain many physical attributes like product quality and operating modes that can help accurately identify faults. As shown in Figure 1a, the shallow structure, which only finds one mapping between low-dimensional features and original process data, might not be sufficient to exploit the complex industrial natures. Fortunately, deep learning-based methods can deal with such cases via hierarchies [11,12,13,14,15]. Unfortunately, most deep network-based methods have apparent deficiencies. Firstly, the very tricky (hyper-)parameter tuning strategy makes deep networks theoretically unexplainable because of too many interfering factors. Secondly, some models require a huge amount of training data; however, fault data are not always sufficient due to the high collecting and labeling costs. Further, they also consume prodigious time and computing resources (such as distributed computers and GPU facilities) during the training process, limiting their wide application in many practical situations. Inspired by the recent advances in deep learning, we conjecture that, if we leverage the hierarchy into other learning models, we might be able to achieve competitive fault diagnosis performance but with fewer of the aforementioned deficiencies.

Currently, hierarchical learning has been developed and applied to a diverse range of practical tasks with much success. Instead of learning a single mapping, hierarchical learning trends to learn multiple layers of mapping, as shown in Figure 1b. There are some efforts to extend shallow models to hierarchical models. For example, Yao et al. [16] introduced a deep discriminative sparse representation learning framework with a deep architecture for machinery fault diagnosis. Chen et al. [17] proposed a deep PCA-based fault diagnosis method for the electrical drive in high-speed trains. Particularly, along with the subspaces obtained by shallow PCA, they continue individually decomposing them until they reach the *n*-th (n≥2)-order subspaces. The higher-order subspace could mine more implicit information about fault data. As the layer number increases, however, the computation cost will exponentially increase because their model has to achieve $2^{n}$ subspaces at the *n*-th layer. Deng et al. [18] stacked multiple Kernel PCA models for layer-wise features, where the output (score vector) of the previous KPCA layer is used as the input for the next KPCA layer. Ren et al. [19] stacked an autoencoder on NMF. The first layer tries to provide the nonlinear interpretation of process data and the second layer achieves dimensionality reduction. Despite of their effectiveness, [18,19] still require tricky parameter/model-structure determination for the nonlinearity interpretation.

Based on the above observations and inspired by the recent advance in deep learning, this paper presents a hierarchical matrix factorization-based method (HMF) for industrial process fault diagnosis, by extending popular NMF to a hierarchical model. The contributions of our work are as follows:

- This paper proposes an HMF for fault diagnosis. The presented model consecutively decomposes fault data, cascading for several hierarchies. The middle hierarchies are expected to learn intrinsic characteristics of processes, the final hierarchy is expected to achieve high-level discriminative features of process data.
- Further, this paper extends HMF to the nonlinear version, referring to nonlinear HMF (NHMF). It adopts activation functions as the nonlinear transformer to describe the nonlinearities in practical industrial processes. Unlike traditional kernel-based methods, this strategy allows our model to avoid tricky kernel parameter tuning.
- The experimental results on the Multi-Phase Flow Process verify the effectiveness of the hierarchies and the nonlinear transformations.

The remainder of this paper is structured as follows. Section 2 introduces the construction of HMF and NHMF. In Section 3, a case study on a practical industrial process is carried out to validate the effectiveness of the proposed method. Our conclusions are drawn in Section 4.

## 2. Preliminary

NMF has gained much attention since it satisfies the psychological and physiological evidence for the part-based learning strategy in human brains [20]. Specifically, NMF discovers the non-negative low-dimensional features $Y^{+}\in {ℜ}^{k\times N}$ of non-negative original process data $X^{+}\in {ℜ}^{d\times N}$ with one mapping $U^{+}\in {ℜ}^{k\times d}$:

(1) $$\min_{U^{+},Y^{+}}\left|\right|X^{+}-U^{+}Y^{+}{\left|\right|}_{F}^{2}$$

where *N* and *d* denote the item number and dimensionality of process data, respectively. *k* is the expected feature dimensionality. $A^{+}$ demotes that matrix A only contains positive elements. It should be noted that this problem is not convex and its suboptimal solutions can be obtained by an iterative multiplicative strategy:

(2) $$\begin{array}{cc} U_{ik} & \leftarrow U_{ik}\frac{{\left(XY^{T}\right)}_{ik}}{{\left(UYY^{T}\right)}_{ik}}\\ Y_{ik} & \leftarrow Y_{kj}\frac{{\left(U^{T}X\right)}_{kj}}{{\left(U^{T}UY\right)}_{kj}} \end{array}$$

where + is omitted for simplicity.

Researchers usually make improvements on NMF to meet the requirements of practical fault diagnosis problems, such as sparseness [21], and geometry preservation [22]. Reference Yang et al. [23] embedded the fault’s prior information into the traditional NMF to enhance its diagnostic performance on diesel engines. Reference Yi et al. [24] adopted the kernel trick on NMF. The Gaussian kernel function was empirically selected to deal with the nonlinearities of industrial processes. In their work, they also introduced the False Nearest Neighbors algorithm into NMF to reduce the fault diagnosis time and space costs. However, they only found one mapping between low-dimensional features and original process data, which might not be sufficient to exploit the complex industrial nature. Motivated by the recent progress of deep learning, we extend the standard NMF to a hierarchical mode, which can automatically learn high-level, discriminative features of industrial process data. We build our discriminative hierarchical feature learning scheme based on the NMF structure; thus, the proposed HMF is still an NMF-based method.

## 3. Methodology

Similar to previous fault diagnosis works, our goal is to obtain a representative feature matrix Y of the original process fault data X. A matrix U serves as the mapping function between Y and X. Industrial process data we wish to analyze often have intrinsic attributes. Leveraging the intrinsic attributes may help accurately identify faults. We conjecture that, if we consecutively map the original process data into several intermediate feature spaces, we may discover rich process attributes and finally achieve discriminant features.

### 3.1. Hierarchical Matrix Factorization

**Figure 2:**
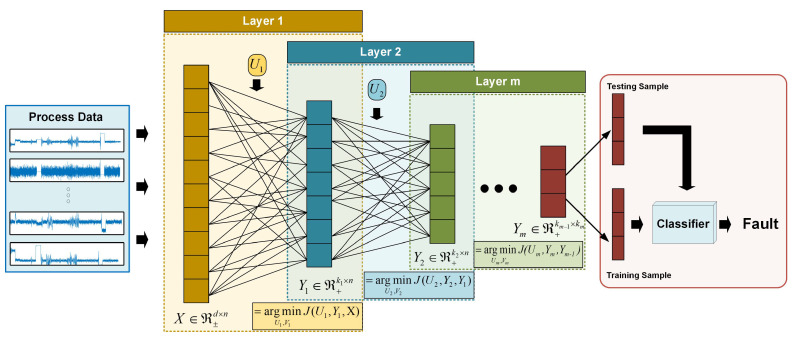
Illustration of the HMF-based fault diagnosis.

Following standard NMF, the hierarchical matrix factorization (HMF) extracts features by consecutively decomposing an original data matrix $X^{+}\in {ℜ}^{d\times N}$ into m+1 layered structures:

(3) $$\begin{array}{c} \min_{U_{1}\cdots U_{m},Y_{m}}\frac{1}{2}\left|\right|X^{+}-U_{1}^{+}\underset{Y_{1}^{+}}{\underbrace{U_{2}^{+}\underset{Y_{2}^{+}}{\underbrace{U_{3}^{+}\cdots \underset{Y_{m-1}^{+}}{\underbrace{U_{m}^{+}Y_{m}^{+}}}}}}}{\left|\right|}_{F}^{2} \end{array}$$

where $\left|\right|\cdot {\left|\right|}_{F}^{2}$ denotes the Frobenius norm. $U_{1}^{+}\in {ℜ}^{d\times k_{1}}$, $U_{m}^{+}\in {ℜ}^{k_{m-1}\times k_{m}}$ and $Y_{m}^{+}\in {ℜ}^{k_{m}\times N}$. As shown in Figure 2, in this model, each hierarchy is expected to be automatically related to a distinct attribute and is assigned the implicit feature accordingly. Finally, the last layer is able to find the high-level features by modeling the complex process attributes.

Since practical process data usually include negative elements, we allow X and U in Equation (3) to have negative parts while enforcing that Y only contains positive elements. This strategy still retains the part-based interpretability [25]. Therefore, the objective function of Equation (3) is formulated as follows:

(4) $$\begin{array}{c} J=\min_{U_{1}\cdots U_{m},Y_{m}}\frac{1}{2}{∥X^{\pm }-\prod_{i=1}^{m}U_{i}^{\pm }Y_{m}^{+}∥}_{F}^{2}\;\;\;s.t.Y_{m}^{+}\geq 0 \end{array}$$

where $A^{\pm }$ demotes that matrix A contains both the positive and negative elements. Equation (4) is a non-convex problem but we can adopt an alternative strategy to solve it, i.e., updating one factor while keeping others fixed. For simplicity, we omit the ± and + in the following equations.

**Updating $U_{i}$ while keeping others fixed.** Obviously, Equation (4) is the following sum of squared residuals:

(5) $$\begin{array}{cc} \; & J=\frac{1}{2}{∥X-\prod_{i=1}^{m}U_{i}Y_{m}∥}_{F}^{2}\\ \; & =tr({\mathrm{XX}}^{T}-2X^{T}\prod_{i=1}^{m}U_{i}Y_{m}+Y_{m}^{T}\prod_{i=1}^{m}U_{i}^{T}\prod_{i=1}^{m}U_{i}Y_{m}) \end{array}$$

We set $\frac{\partial J}{\partial U_{i}}=0$ and give ourselves the following updating rule for $U_{i}$:

(6) $$U_{i}\leftarrow {\left[{\left(\prod_{s=1}^{i-1}U_{s}\right)}^{T}\prod_{s=1}^{i-1}U_{s}\right]}^{-1}{\left(\prod_{s=1}^{i-1}U_{s}\right)}^{T}XY_{i}^{T}{\left(Y_{i}Y_{i}^{T}\right)}^{-1}$$

**Updating $Y_{i}$ while keeping others fixed.** We employ the Lagrange multiplier $\Phi_{i}$ for the non-negativity constraint on $Y_{i}$ and the Lagrange function related to $Y_{i}$ is written as

(7) $$\begin{array}{cc} \; & L(Y_{i})=\\ \; & Tr\left(-2X^{T}\prod_{s=1}^{i-1}U_{s}Y_{i}+Y_{i}^{T}\prod_{i=1}^{i-1}U_{s}^{T}\prod_{s=1}^{i}U_{i}Y_{i}\right)-Tr\left(\Phi_{i}Y_{i}\right) \end{array}$$

Let its gradient be equal to zero; from the complementary slackness condition, we can obtain

(8) $$\begin{array}{c} \left[-{\left(\prod_{s=1}^{i}U_{s}\right)}^{T}X+{\left(\prod_{s=1}^{i}U_{s}\right)}^{T}\prod_{s=1}^{i}U_{s}Y_{i}\right]⊙Y_{i}=\Phi_{i}⊙Y_{i}=0 \end{array}$$

where ⊙ denotes the dot-product.

This is a fixed-point equation that the limiting solution must satisfy at the convergence of $Y_{i}^{\left(\infty \right)}=Y_{i}^{\left(t+1\right)}=Y_{i}^{\left(t\right)}=Y_{i}$, i.e.,

(9) $$\begin{array}{cc} \; & Y_{i}\leftarrow \\ \; & Y_{i}⊙\sqrt{\frac{{\left[{\left(\prod_{s=1}^{i-1}U_{i}\right)}^{T}X\right]}^{pos}+{\left[{\left(\prod_{s=1}^{i-1}U_{i}\right)}^{T}\prod_{s=1}^{i-1}U_{i}\right]}^{neg}Y_{i}}{{\left[{\left(\prod_{s=1}^{i-1}U_{i}\right)}^{T}X\right]}^{neg}+{\left[{\left(\prod_{s=1}^{i-1}U_{i}\right)}^{T}\prod_{s=1}^{i-1}U_{i}\right]}^{pos}Y_{i}}} \end{array}$$

where $A^{pos}$ denotes a matrix that only contains all the positive elements; $A^{neg}$ denotes a matrix that only contains all the negative elements:

(10) $$A^{pos}=\frac{|A|+A}{2},\;\;\;\;A^{neg}=\frac{|A|-A}{2}$$

By iteratively updating factors U and Y with Equations (6) and (9), we can get the sub-optimal solution to the formulated Equation (4).

Pre-training has been successfully employed in previous deep learning works and it can greatly reduce the training time [12]. We also followed this tactic to have an initial approximation of each layer and, thus, expedite the approximation of $U_{i}$ and $Y_{i}$ in the HMF and NHMF. To be specific, the original data matrix X is firstly decomposed into $U_{1}\in {ℜ}_{\pm }^{d\times k_{1}}$ and $Y_{1}\in {ℜ}_{0,+}^{k_{1}\times n}$ using conventional semi-NMF [13]. Further, $Y_{1}$ is decomposed into $U_{2}\in {ℜ}_{\pm }^{k_{1}\times k_{2}}$ and $Y_{2}\in {ℜ}_{0,+}^{k_{2}\times n}$, continuing to do so until all layers have been initialized. Afterward, $U_{i}$ and $Y_{i}$ are iteratively fine-tuned via Equations (6)–(9) until convergence. The stop criterion of the algorithms could be the maximum iteration or small-loss value.

The computational complexity and space complexity are calculated as follows. We need O(dNk) for Equation (6) to update $U_{i}$ and $O(dNk+\left(d+N\right)k^{2})$ for Equation (9) to update $Y_{i}$, where $k=max\{k_{i}\}$. Therefore, to optimize a model with *m* layers and *t* iterations, the overall computational complexity is $O(\left(dNk+\left(d+N\right)k^{2}\right)mt)$.

### 3.2. Nonlinear Hierarchical Matrix Factorization

Practical industrial processes usually exhibit nonlinearity due to their complex system mechanism, coupling sensors and varying operating conditions. The nonlinearity brings difficulties to the HMF since it can only learn the low-dimensional features by linear mapping. The failure to describe the nonlinear correlations may lead to unsatisfactory fault diagnosis performance.

From the mathematical point of view, to bring the nonlinearity to the HMF, one can utilize a nonlinear function in each layer of representations:

(11) $$Y_{i-1}=\varphi \left(U_{i}Y_{i}\right)$$

where φ denotes a nonlinear function. Popular choices could be kernel functions. The kernel trick, however, usually requires cumbersome parameter determinations. Therefore, activation functions are introduced to the HMF. In this situation, the nonlinear extension of Equation (4) is formulated as

(12) $$J_{non}=\frac{1}{2}{∥X^{\pm }-U_{1}^{\pm }\varphi \left(\cdots \varphi \left(U_{m}^{\pm }Y_{m}^{+}\right)\right)∥}_{F}^{2}\;\;\;s.t.Y_{m}\geq 0$$

We can use the gradient descent optimizations to minimize this cost function and the derivative for each factor needs to be computed.

Particularly, when i=1, this model is equivalent to the shallow one:

(13) $$\min_{U_{1},Y_{1}}\frac{1}{2}{∥X-U_{1}\varphi \left(Y_{1}\right)∥}_{F}^{2}$$

and the derivation of $J_{non}$ with respect to $Y_{1}$ is written as

(14) $$\begin{array}{cc} \frac{\partial J_{non}}{\partial Y_{1}} & =\frac{1}{2}\frac{\partial Tr\{-2X^{T}U_{1}\varphi \left(Y_{1}\right)+{\left[U_{1}\varphi \left(Y_{1}\right)\right]}^{T}U_{1}\varphi \left(Y_{1}\right)\}}{\partial \varphi (Y_{1})}⊙\nabla \varphi \left(Y_{1}\right)\\ \; & =U_{1}^{T}U_{1}\varphi \left(Y_{1}\right)-U_{1}^{T}X⊙\nabla \varphi \left(Y_{1}\right)\\ \; & =U_{1}^{T}\left[U_{1}\varphi \left(Y_{1}\right)-X\right]⊙\nabla \varphi \left(Y_{1}\right) \end{array}$$

the derivation of $J_{non}$ with respect to $U_{1}$ is written as

(15) $$\begin{array}{cc} \frac{\partial J_{non}}{\partial U_{1}} & =\frac{1}{2}\frac{\partial Tr\{-2X^{T}U_{1}\varphi \left(Y_{1}\right)+{\left[U_{1}\varphi \left(Y_{1}\right)\right]}^{T}U_{1}\varphi \left(Y_{1}\right)\}}{\partial \varphi (U_{1})}\\ \; & =\left[U_{1}\varphi \left(Y_{1}\right)-X\right]\varphi \left(Y_{1}^{T}\right) \end{array}$$

In order to compute the derivative of $Y_{i}$(i≥2), we use the chain rule:

(16) $$\begin{array}{cc} \frac{\partial J_{non}}{\partial Y_{i}} & =U_{i}^{T}\frac{\partial J_{non}}{\partial U_{i}Y_{i}}\\ \; & =U_{i}^{T}\left[\frac{\partial J_{non}}{\partial \varphi (U_{i}Y_{i})}⊙\nabla \varphi \left(U_{i}Y_{i}\right)\right]\\ \; & =U_{i}^{T}\left[\frac{\partial J_{non}}{\partial Y_{i-1}}⊙\nabla \varphi \left(U_{i}Y_{i}\right)\right] \end{array}$$

Similarly, for $U_{i}$(i≥2), we obtain

(17) $$\begin{array}{cc} \frac{\partial J_{non}}{\partial U_{i}} & =\frac{\partial J_{non}}{\partial U_{i}Y_{i}}Y_{i}^{T}\\ \; & =\left[\frac{\partial J_{non}}{\partial \varphi (U_{i}Y_{i})}⊙\nabla \varphi \left(U_{i}Y_{i}\right)\right]Y_{i}^{T}\\ \; & =\left[\frac{\partial J_{non}}{\partial U_{i-1}}⊙\nabla \varphi \left(U_{i}Y_{i}\right)\right]Y_{i}^{T} \end{array}$$

With these derivatives, gradient descent optimizations can be utilized to minimize the cost function with respect to each layer of $U_{i}$ and $Y_{i}$.

(18) $$\begin{array}{cc} Y_{i} & \leftarrow Y_{i}-\eta \frac{\partial J_{non}}{\partial Y_{i}}\\ U_{i} & \leftarrow U_{i}-\eta \frac{\partial J_{non}}{\partial U_{i}} \end{array}$$

where η is the learning rate.

By iteratively updating factors U and Y with Equation (18), we can get the sub-optimal solution to the formulated equation, Equation (12). Similar to HMF, pre-training can greatly expedite the approximation procedure and we also use conventional semi-NMF to initialize each layer of this model. Afterward, each layer is fine-tuned via Equation (18) until convergence.

### 3.3. Fault Identification

As shown in Figure 2, since both HMF and NHMF are unsupervised models, all samples $X=[X^{train};X^{test}]$ need to be fed into our model to learn the fault features $Y_{m}=\left[Y^{train};Y^{test}\right]$. Further, $Y_{m}$ is divided into $Y^{train}$ and $Y^{test}$. $Y^{train}$ is utilized to train a simple classifier C while the fault type of $Y^{test}$ can be predicted with the classifier C. In this work, we adopt the *K*-Nearest Neighbor (KNN) classifier for its simplicity and efficiency. To be specific, the KNN assigns $y_{i}^{test}$ to the fault type with the following rules:

(19) $$\begin{array}{cc} if, & \;\;i_{0}=\min_{i}∥y_{i}^{test}-y_{i}^{train}∥,\\ then, & \;\;label\left(y_{i}^{test}\right)=label\left(y_{i0}^{train}\right) \end{array}$$

If a new datum $x^{*}$ is coming, we can either use HMF or NHMF to project it to learn its feature $y^{*}$. This can be achieved via basic matrix reconstruction:

(20) $$y^{*}\approx {\left[U_{1}U_{2}\ldots U_{m}\right]}^{†}x^{*}$$

for the linear model, and

(21) $$y^{*}\approx \varphi^{-1}\left(U_{m}^{†}\left(\cdots \left(U_{2}^{†}\varphi^{-1}\left(U_{1}^{†}x^{*}\right)\right)\right)\right)$$

for the nonlinear model, where † denotes the Moore–Penrose pseudo-inverse.

## 4. Case Study

To verify the effectiveness of the proposed methods, this section provides the experimental results and discussion on the Multiple Phase Flow process (MPF) (http://www.mathworks.com/matlabcentral/fileexchange/50938-a-benchmark-case-for-statistical-process-monitoring-cranfield-multiphase-flow-facility (accessed on 10 June 2024)).

### 4.1. Data Description

**Figure 3:**
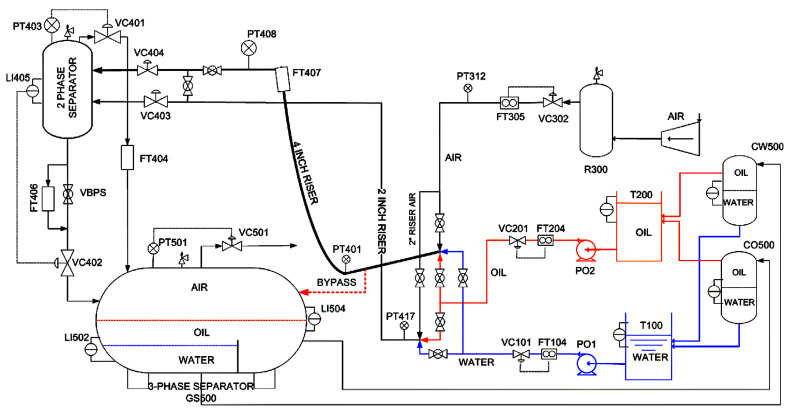
Diagram of the Multiple Phase Flow process [26].

The MPF is utilized to verify the effectiveness of the proposed models. It was designed by Cranfield University to provide a controlled and measured flow rate of water, oil and air to a pressurized system whose diagram is shown in Figure 3. In the MPF process, there are 24 measurements to describe the condition of this process. All the data were captured at a sampling rate of 1 Hz. As summarized in Table 1, we select five different conditions as the dataset, which include the normal condition, air line blockage, water line blockage, top separator input blockage and open direct bypass. A total of 50% of the samples are utilized as the training dataset while others are the testing dataset. In particular, these fault data are collected under changing operational conditions instead of in the steady-state regime. The multimode and nonlinearity characteristics, together with the system size of the MPF, make this case a desirable benchmark.

### 4.2. Comparison with Shallow Methods

In this work, the shallow model is a type of machine learning algorithm with only one layer of composition. The linear models include Principal Component Analysis (PCA) [27], Linear Discriminant Analysis (LDA) [28], Sparse Discriminant Analysis (SDA) [29] and Non-negative Matrix Factorization (NMF) [20], while the nonlinear models include Kernel Principal Component Analysis (KPCA) [30], Sparse Exponential Discriminant Analysis (SEDA) [31] and Kernel Non-negative Matrix Factorization (KNMF) [32]. For all methods, the final representation dimensionality *k* was set to 5, except LDA, which was set to 4. For SDA and SEDA, their parameters were set as recommended by the author in the original paper. For the kernel-based methods, the Gaussian kernel was empirically set to 1.3. The offset and degree parameters of the polynomial kernel in PNMF were set to 1 and 20, respectively. For both the HMF and NHMF, we set the layer number *m* to 3 and their size was set to 19, 12 and 5 for the first to third layers. Moreover, the nonlinear function for NHMF is selected as the tanh function.

We report the performance of different methods related to each fault class, where the performance is evaluated by the True Positive Rate (TPR) and Positive Predictive Value (PPV):

(22) $$\begin{array}{cc} TPR & =n_{i}^{t}/n_{i}\times 100\%,\;\;\;Avg\left(TPR\right)=\sum_{i=1}^{C}\times TPR\left(i\right)/n\\ PPV & =n_{i}^{t}/n_{i}^{p}\times 100\%,\;\;\;Avg\left(PPV\right)=\sum_{i=1}^{C}\times PPV\left(i\right)/n \end{array}$$

where *C* denotes the total number of fault types. *n* is the number of total samples. $n_{i}^{t}$ and $n_{i}$ are the correctly diagnosed sample number and total sample number in *i*-th fault type, respectively. $n_{i}^{p}$ is the number of predicted samples as the *i*-th fault. Specifically, TPR measures the accuracy of the models and PPV measures the precision of the models. Larger values denote better performance.

The diagnosis performance with respect to TPR and PPV is summarized in Table 2. There are some interesting observations in this table. (1) Comparing the results between PCA and KPCA, between LDA/SDA and SEDA and between NMF and KNMF, models with kernel or activation functions achieve more promising diagnosis performance. This may be attributed to the nonlinearity of the MPF process. (2) During the grid search procedure for kernel parameters, we find that some parameter combinations even decrease the fault diagnosis performance of KPCA and KNMF. How to determine proper parameters is very tricky in practical cases, whereas NHMF only requires the selection of activation functions. (3) Both HMF and NHMF perform better than the shallow NMF. To be specific, the average TPR and PPV of HMF are 5.33% and 4.13% higher than those of the best competitors; the average TPR and PPV of NHMF are 7.58% and 6.70% higher than those of the best competitors.

**Figure 4:**
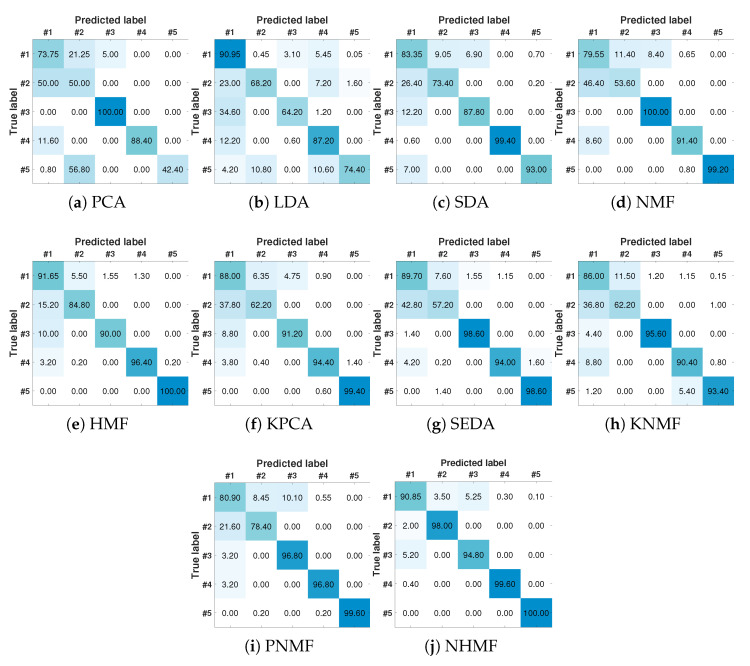
The confusion matrices of different methods.

For more detailed results of diagnosis performance, the confusion matrices of different methods are provided in Figure 4. Class #1 denotes the normal condition of TPF while #2–#5 denote air line blockage, water line blockage, top separator input blockage and open direct bypass, respectively. It is clear that the results of NHMF have the most obvious diagonal block structure among these five methods. Namely, NHMF works well for all four fault types and one normal condition. HMF performs well on faults #3, #4 and #5 while it has a relevant higher misclassification error on class #1 and class #2. Shallow models perform even worse because they fail to exploit complex attributes of industrial processes.

### 4.3. Comparison with Deep Models

As for deep models, our work mainly focuses on deep neural networks that consist of multiple fully connected layers: an input layer, multiple hidden layers (at least two) and a single output layer. In this experiment, we selected two typical deep neural network-based fault diagnosis methods as the competitors, i.e., Stacked Auto-Encoder (SAE) and Deep Boltzmann Machine (DBN). A total of four layers were constructed in SAE and DBN whose neural numbers from the first layer to the fourth layer were set to 24, 19, 12 and 5, respectively.

**Figure 5:**
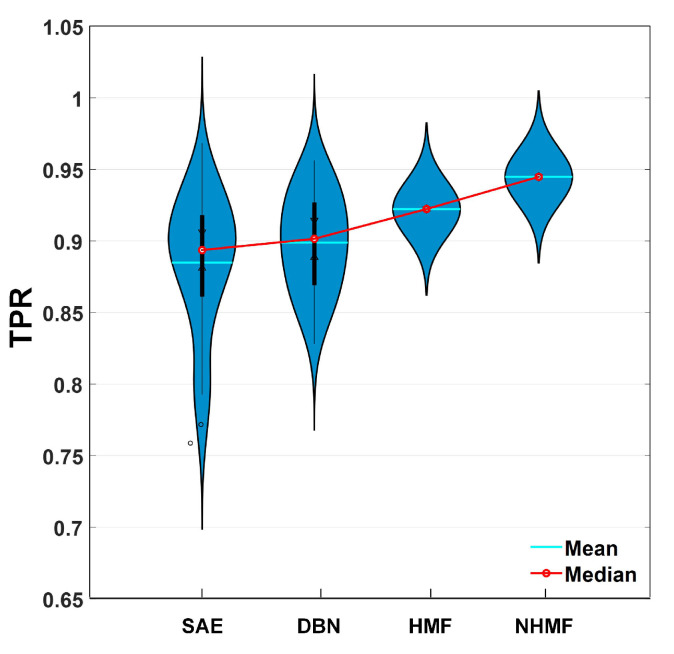
Diagnosis performance of deep models and our models in terms of TPR.

The experimental results are reported in Table 3. Compared with Table 2, all the hierarchical models achieve better diagnosis performance, with approximately 90% TPR and PPV. This fact shows the effectiveness of the deep learning strategy. We also provide the violin plots of the obtained results in Figure 5. Only the results in terms of TPR are provided. Similar observations also can be seen in terms of PPV. Generally speaking, the performance of deep neural networks varies in a large range (80%, 96%). This may be attributed to the limited training samples and randomized initialization. We cannot deny that the potential power of deep neural networks is extremely strong. However, their powerful performance depends heavily on some tricky operations. If they are not well-tuned, they may also achieve an unsatisfactory performance, whereas our model can achieve a relatively stable and desirable diagnosis performance.

### 4.4. Time Consumption Analysis

**Figure 6:**
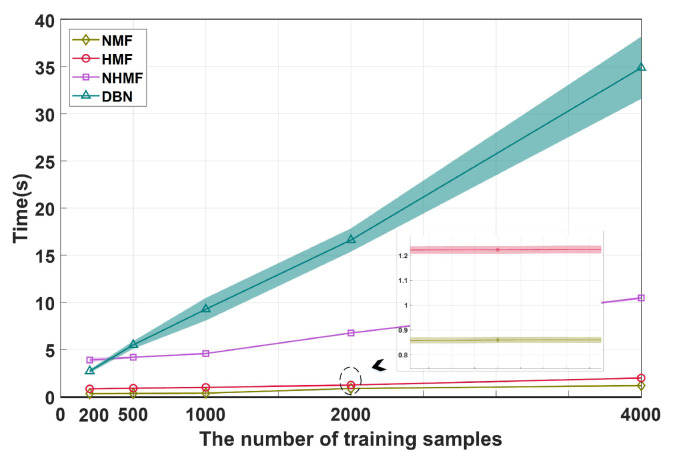
Average time cost comparison of NMF, HMF, NHMF and DBN.

In this experiment, we compared the average time consumption of the HMF and NMHF with DBN and NMF. Specifically, we varied the number of training samples between 200, 500, 1000, 2000 and 4000 and each algorithm was performed 50 times to avoid randomness. The layer sizes of both the HMF and NHMF were set to 19-12-5. Their maximum iteration was set to 50, since they converge very fast. The configurations of DBN and NMF were set as previously mentioned. Figure 6 shows the results where the shadow denotes variance. We only report the time of DBN since similar observations can be found in SAE. Clearly, from this figure, shallow architecture (NMF) generally takes the least computational time among these competitors due to its simple structure. The time cost of HMF is slightly higher than that of NMF but it is still within an acceptable range. NHMF costs much more time because the derivation of nonlinear functions brings computational complexity during optimization. However, it is better than DBN, which costs the most prodigious time during the training procedure.

### 4.5. Effectiveness of Hierarchies

To show the effectiveness of the layer-wise structure of HMF and NHMF, we provide the diagnosis results obtained by the shallow model and hierarchical models. The configuration of NMF is similar to the previous one. For the two-layer-structured HMF and NHMF, their layer size numbers were set to 19 and 5 for the first and second layers while, for the three-layer-structured HMF and NHMF, their layer size numbers were set to 19, 12 and 5 from the first to last layer. As shown in Table 4 and Table 5, the deeper structured models achieve higher scores in both TPR and PPV. To be specific, the average TPR of shallow-model NMF is 82.80% while those of two-layer models are 89.78% and 88.50%. Furthermore, the three-layer-structured HMF and NHMF outperform both the shallow model and the two-layer models by exceeding 90% TRP and PPV in the experiments. This fact demonstrates that a relatively deeper layer can truly yield better diagnosis performance. However, the increasing layers also may even decrease the performance because the limited data make the deep model under-fitted. Unfortunately, similar to other deep models, how to precisely decide the number of HMFs and NHMFs is still an open problem in research because it depends on the quality of available data. Some empirical strategies can help the decision, such as trial and error, heuristic search and exhaustive search. In this paper, we suggest (2,4) layers for the datasets whose scales are similar to the MPF process.

### 4.6. Parameter Study on Hierarchical Structure

**Figure 7:**
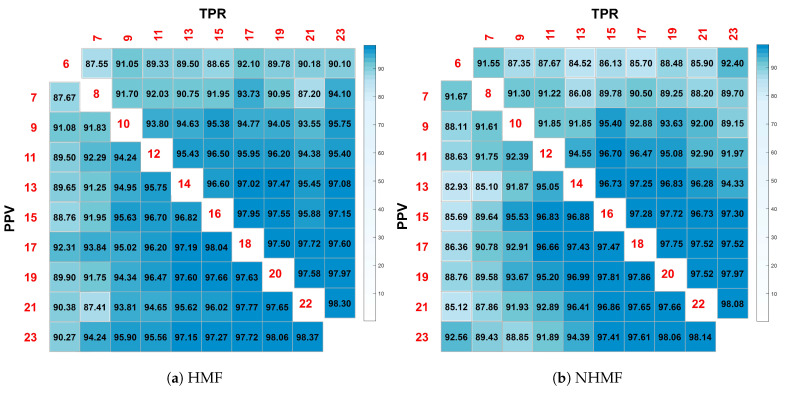
The performance heat maps when models adopt different layer sizes: (**a**) HMF and (**b**) NHMF.

Next, we proceeded to evaluate the influences of the layer size. To achieve this goal, we constructed a two-layer HMF and NHMF. The size of their first layer varied from 7 to 23 and the size of the second layer varied from 6 to 22, all with the interval 2. Figure 7 illustrates the performance heat map when HMF and NHMF adopt different layer sizes. In Figure 7, the number on the diagonal is the size of the last layer while the number on the horizontal and vertical axes is the size of the first layer. It should be noted that the size of the first layer is larger than that of the last layer. Clearly, as the layer size increases, both the TPR and PPV scores are getting higher. To balance the performance and model size, we may suggest the size of the *m*-th layer to be ${\left(\lambda \right)}^{m}d$, where λ∈ (0.6,0.8).

### 4.7. Convergence Analysis

**Figure 8:**
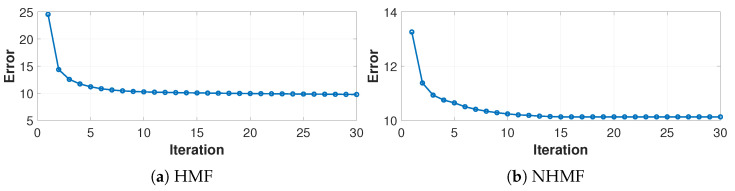
The convergence curves of (**a**) HMF and (**b**) NHMF.

As discussed in previous sections, we adopted iterative updating rules to obtain the local optima of HMF and NHMF. Figure 8 experimentally illustrates the convergence of our models on the TPP dataset. As we can see, the loss value of both HMF and NHMF monotonically decreases with the increase of iterations and converges fast. Specifically, HMF converges to be stable within 10 iterations while NHMF converges to be stable within 15 iterations.

### 4.8. Visualization of the Learned Feature Matrices

**Figure 9:**
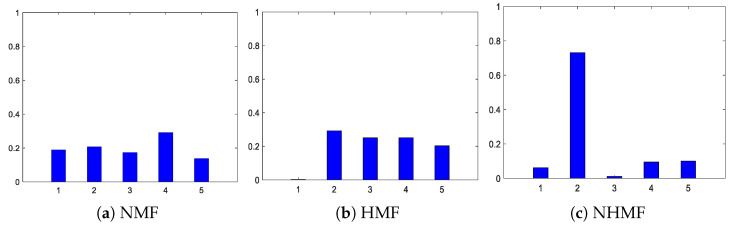
The weight vectors of Y. (**a**) NMF, (**b**) HMF and (**c**) NHMF.

The learned fault features matrices Y are visualized in Figure 9, where the horizontal axis denotes the learned feature and the vertical axis is the weight value. It should be noted that all weight values in the matrix Y are normalized to 0-1. It reveals that the five weight values extracted from the NMF are nearly indistinguishable from the remaining values, thus leading to possibly learning some indiscriminate features. In contrast, the discrimination between the weight values of HMF and NHMF is notably enhanced, contributing to the superior fault diagnosis performance of the proposed method.

## 5. Conclusions

This paper presented a hierarchical matrix factorization-based fault diagnosis method. HMF consecutively decomposes the original process data into several intermediate spaces so that it can automatically learn process attributes, allowing for better feature discrimination and interpretability. Further, to provide nonlinear interpretability for practical industrial processes, the HMF is extended to a nonlinear case with the aid of activation functions. The experiment results on a practical process demonstrate that, by adding intermediate hierarchies in the conventional shallow model, the presented HMF is able to learn high-level, discriminative features of industrial process data. With activation functions, the NHMF is able to deal with nonlinearities existing in practical industrial processes without tricky parameter tuning and achieve better diagnosis performance. Both of them outperformed the considered range of typical powerful fault diagnosis methods, consuming less computing time than the considered deep models.

However, the proposed approach still has some limitations. Firstly, the proposed method may be a bit sensitive to outliers. When the training data are contaminated heavily because of noise and outliers, the proposed method may result in a degenerated classification performance. Secondly, it complies with the underlying assumptions of balanced data distributions. When the data are imbalanced, the proposed method may tend to strongly favor the majority fault class and detect the minority fault class at extremely low rates, leading to poor fault diagnosis performance. Future work will focus on designing a more robust classification framework and considering imbalanced data distributions to achieve better diagnosis performance.

## Figures and Tables

**Table 1:** The description of the TPF process.

Class	Type	Description	#Samples
#1	Normal condition	-	4000
#2	Fault	Air line blockage	1000
#3	Fault	Water line blockage	1000
#4	Fault	Top separator input blockage	1000
#5	Fault	Open direct bypass	1000

**Table 2:** The diagnosis performance comparison in terms of TPR and PPV.

	**Method**	**avg.TPR**	**avg.PPV**		**Method**	**avg.TPR**	**avg.PPV**
	**PCA(5)**	71.98%	79.95%		**KPCA(Gaussian)**	87.40%	87.21%
	**LDA(4)**	82.23%	83.21%		**SEDA(Default)**	88.40%	88.05%
**Linear**	**SDA(4)**	85.88%	86.41%	**Nonlinear**	**KNMF(Gaussian)**	85.70%	86.05%
	**NMF(5)**	82.80%	83.29%		**KNMF(Polynomial)**	86.90%	88.25%
	**HMF(19-12-5)**	**92.23**%	**92.34**%		**NHMF(19-12-5)**	**94.48**%	**94.45**%

**Table 3:** The diagnosis performance comparison of deep models.

	SAE	DBN	HMF	NHMF
**avg. TPR**	88.49%	89.88%	92.23%	94.48%
**avg. PPV**	88.45%	89.98%	92.34%	94.45%

**Table 4:** The performance comparison when NHMF adopts different layers.

	NMF		NHMF							
	**Shallow** **(5)**		**2-Layer** **(19-5)**		**3-Layer** **(19-12-5)**		**4-Layer** **(20-15-10-5)**		**5-Layer** **(22-20-15-10-5)**	
	**TPR**	**PPV**	**TPR**	**PPV**	**TPR**	**PPV**	**TPR**	**PPV**	**TPR**	**PPV**
**#1**	79.55%	85.26%	85.95%	90.57%	90.85%	97.95%	93.05%	96.18%	92.20%	95.20%
**#2**	53.60%	54.03%	94.00%	81.74%	98.00%	87.50%	94.80%	84.79%	88.20%	84.48%
**#3**	100.00%	74.85%	72.20%	68.89%	94.80%	81.87%	91.20%	91.38%	95.20%	89.81%
**#4**	91.40%	96.41%	98.00%	97.42%	99.60%	98.81%	99.20%	97.83%	98.00%	96.27%
**#5**	99.20%	100.00%	100.00%	100.00%	100.00%	99.60%	99.80%	99.80%	100.00%	99.60%
**Avg.**	**82.80%**	**83.29%**	**88.50%**	**88.79%**	**94.48%**	**94.95%**	**94.65%**	**94.81%**	**93.78%**	**93.87%**

**Table 5:** The performance comparison when HMF adopts different layers.

	NMF		HMF							
	**Shallow** **(5)**		**2-Layer** **(19-5)**		**3-Layer** **(19-12-5)**		**4-Layer** **(20-15-10-5)**		**5-Layer** **(22-20-15-10-5)**	
	**TPR**	**PPV**	**TPR**	**PPV**	**TPR**	**PPV**	**TPR**	**PPV**	**TPR**	**PPV**
**#1**	79.55%	85.26%	89.00%	91.24%	91.65%	92.81%	88.60%	88.78%	89.50%	92.55%
**#2**	53.60%	54.03%	74.00%	71.43%	84.80%	79.25%	79.20%	74.86%	77.80%	75.68%
**#3**	100.00%	74.85%	96.20%	89.91%	90.00%	93.56%	79.40%	80.36%	97.20%	87.88%
**#4**	91.40%	96.41%	95.20%	93.15%	96.40%	94.88%	96.60%	96.60%	95.20%	90.32%
**#5**	99.20%	100.00%	96.80%	99.79%	100.00%	99.80%	96.00%	99.79%	93.40%	98.94%
**Avg.**	**82.80%**	**83.29%**	**89.78%**	**89.90%**	**92.23%**	**92.34%**	**88.20%**	**88.34%**	**90.20%**	**90.38%**

## Data Availability

Data are contained within the article.
